# Critical care management and outcome of severe *Pneumocystis *pneumonia in patients with and without HIV infection

**DOI:** 10.1186/cc6806

**Published:** 2008-01-25

**Authors:** Xavier Monnet, Emmanuelle Vidal-Petiot, David Osman, Olfa Hamzaoui, Antoine Durrbach, Cécile Goujard, Corinne Miceli, Patrice Bourée, Christian Richard

**Affiliations:** 1 AP-HP, Hôpital de Bicêtre, service de réanimation médicale, 78, rue du Général Leclerc, Le Kremlin-Bicêtre, F-94270, France; 2 Univ Paris-Sud, Faculté de médecine Paris-Sud, EA 4046, 78, rue du Général Leclerc, Le Kremlin-Bicêtre, F-94270, France; 3 AP-HP, Hôpital de Bicêtre, service de néphrologie, 78, rue du Général Leclerc, Le Kremlin-Bicêtre, F-94270, France; 4 AP-HP, Hôpital de Bicêtre, service de médecine interne, 78, rue du Général Leclerc, Le Kremlin-Bicêtre, F-94270, France; 5 Univ Paris-Sud, INSERM, UMR_S 802, 78, rue du Général Leclerc, Le Kremlin Bicêtre, F-94270, France; 6 AP-HP, Hôpital de Bicêtre, service de rhumatologie, 78, rue du Général Leclerc, Le Kremlin-Bicêtre, F-94270, France; 7 AP-HP, Hôpital de Bicêtre, unité des maladies parasitaires, 78, rue du Général Leclerc, Le Kremlin-Bicêtre, F-94270, France

## Abstract

**Background:**

Little is known about the most severe forms of *Pneumocystis jiroveci *pneumonia (PCP) in HIV-negative as compared with HIV-positive patients. Improved knowledge about the differential characteristics and management modalities could guide treatment based on HIV status.

**Methods:**

We retrospectively compared 72 patients (73 cases, 46 HIV-positive) admitted for PCP from 1993 to 2006 in the intensive care unit (ICU) of a university hospital.

**Results:**

The yearly incidence of ICU admissions for PCP in HIV-negative patients increased from 1993 (0%) to 2006 (6.5%). At admission, all but one non-HIV patient were receiving corticosteroids. Twenty-three (85%) HIV-negative patients were receiving an additional immunosuppressive treatment. At admission, HIV-negative patients were significantly older than HIV-positive patients (64 [18 to 82] versus 37 [28 to 56] years old) and had a significantly higher Simplified Acute Physiology Score (SAPS) II (38 [13 to 90] versus 27 [11 to 112]) but had a similar PaO_2_/FiO_2 _(arterial partial pressure of oxygen/fraction of inspired oxygen) ratio (160 [61 to 322] versus 183 [38 to 380] mm Hg). Ventilatory support was required in a similar proportion of HIV-negative and HIV-positive cases (78% versus 61%), with a similar proportion of first-line non-invasive ventilation (NIV) (67% versus 54%). NIV failed in 71% of HIV-negative and in 13% of HIV-positive patients (*p *< 0.01). Mortality was significantly higher in HIV-negative than HIV-positive cases (48% versus 17%). The HIV-negative status (odds ratio 3.73, 95% confidence interval 1.10 to 12.60) and SAPS II (odds ratio 1.07, 95% confidence interval 1.02 to 1.12) were independently associated with mortality at multivariate analysis.

**Conclusion:**

The yearly incidence of ICU admissions for PCP in HIV-negative patients in our unit increased from 1993 to 2006. The course of the disease and the outcome were worse in HIV-negative patients. NIV often failed in HIV-negative cases, suggesting that NIV must be watched closely in this population.

## Introduction

In developed countries, the introduction of the prophylaxis against *Pneumocystis jiroveci *pneumonia (PCP) and of highly active antiretroviral therapy has resulted in a decline of this disease in recent years in patients with HIV infection [1]. However, PCP-induced acute respiratory failure remains a leading cause of intensive care unit (ICU) admission in patients with AIDS [2]. By contrast, the incidence of PCP in patients with predisposing immunodeficiencies other than AIDS is growing [3-5].

The studies that have recently analyzed PCP in patients with and without HIV infection [3-6] did not specifically address the comparison between these populations in the most critical forms of PCP. For instance, when hospitalized in the ICU, at least two thirds of PCP patients need mechanical ventilation [3,7-12], which generally is associated with a very high 'in-hospital' mortality [3,7-11]. Nevertheless, no study has investigated the effect of HIV status on the severe forms of PCP, particularly concerning the effectiveness of mechanical ventilation. This may be particularly important since the lung impairment may be worse in HIV-negative patients.

Thus, we performed this study in order to compare the critical care management and outcome of HIV-positive and HIV-negative patients admitted to our institution for PCP over a period (1993 to 2006) when corticosteroids were systematically administered in severe AIDS-related PCP. Improved knowledge of the different characteristics and outcomes between HIV-negative and HIV-positive patients with PCP could help the physician in managing treatment based on HIV status, particularly as it concerns ventilatory support.

## Materials and methods

### Identification of cases

Our observational study was conducted in a 22-bed medical ICU that receives around 1,000 patients each year and that belongs to a university hospital. This institution provides care for 1,200 to 1,500 HIV-positive patients and for a miscellaneous population of HIV-negative immunocompromised patients, including 2,500 renal transplant recipients (438 renal transplantations from January 1993 to December 1999 and 679 from January 2000 to December 2006). With the agreement of our institutional review board, we retrospectively collected the medical charts of all consecutive PCP patients admitted to our ICU from January 1993 through December 2006. All patients or next of kin were informed at the time of hospitalization that the medical chart could be used for later statistical analysis and gave their consent. For all patients, the diagnosis of PCP was made by the identification of *P. jiroveci *organisms with immunofluorescence, Giemsa, or Gomori-Grocott in specimens of bronchoalveolar lavage, induced sputum, or tracheal aspiration. *P. jiroveci *polymerase chain reaction was not performed. HIV-negative patients were defined by a negative HIV-1 antibody test.

### Collection of data

Recorded data concerned general demographic information; comorbid condition; prehospital use of antiretroviral, prophylactic, and immunosuppressive medications; initial vital signs and laboratory data; organ failures and severity of the disease at admission; associated infections; therapeutic modalities; medications received; time course and modalities for ventilatory support; hospital and ICU lengths of stay; and ICU, 28-day, and 90-day mortality rates.

Severity of illness on admission was assessed by using the Simplified Acute Physiology Score (SAPS) II. For patients admitted before 1995, SAPS I was calculated and converted to SAPS II by using the formula SAPS II = 0.94 + (2.6 × SAPS I). The HIV-positive patients with PCP were classified as 'AIDS' in the chronic disease component of the SAPS. Late non-invasive mechanical ventilation (NIMV) failure was defined by the need for endotracheal intubation that occurred at least 48 hours after NIMV initialization. Acute respiratory failure was defined as a PaO_2_/FiO_2 _(arterial partial pressure of oxygen/fraction of inspired oxygen) of less than or equal to 300 mm Hg [13] or the need for mechanical ventilation. Acute circulatory failure was defined as a systolic blood pressure of less than or equal to 90 mm Hg (or a decrease of greater than or equal to 50 mm Hg in previously hypertensive patients), the need for vasopressive agents (dopamine of greater than or equal to 5 μg/kg per minute or norepinephrine), or an elevated blood lactate level (≥ 2 mmol/L) [14]. The density of *P. jiroveci *organisms was graded as 'many' when foamy alveolar casts were easily visualized on all slides and as 'few' when foamy alveolar casts were not individualized at first-glance examination [13]. Ventilator-associated pneumonia was defined by the association of a clinical suspicion and of positive quantitative cultures of distal pulmonary secretion samples obtained by fiberoptic bronchoscopy of bronchoalveolar lavage fluid (significant threshold of greater than or equal to 10^4 ^colony-forming units per milliliter) or of a protected specimen brush (significant threshold of greater than or equal to 10^3 ^colony-forming units per milliliter) [15].

### Ventilation support

The modalities of ventilation support were not determined by standardized protocol but by the current practice at our department. According to this practice, when use of oxygen did not enable a significant improvement, NIMV was delivered to the patient through a full face mask. In all patients, NIMV was performed with the ventilator set in the pressure-support mode (positive end-expiratory pressure of between 5 and 7 cm H_2_O, pressure support adjusted to obtain an expired tidal volume of 7 to 10 mL/kg of body weight, and a respiratory rate of less than 25 breaths per minute). The FiO_2 _was adjusted to maintain an arterial oxygen saturation of greater than or equal to 90%. The attending physician made the decision to perform endotracheal intubation either as first-line therapy or when NIMV failed. This decision was made without a standardized protocol.

### Statistical analysis

Continuous data are expressed as median (range) and were compared between HIV-positive and HIV-negative patients by using a two-tailed Student *t *test or the Wilcoxon rank sum test as appropriate. Non-continuous dichotomous data were compared between HIV-positive and HIV-negative patients with the χ^2 ^test with Yates correction or with the Fisher exact test as appropriate. For testing the time course of mortality, mortality in patients requiring mechanical ventilation, and the proportion of patients requiring ventilation assistance, we evaluated the linear correlation of those variables with time by using the least squares linear regression method. We performed a multivariate analysis to test the dependence of ICU mortality on each variable by logistic regression, as measured by the estimated odds ratio (OR) with 95% confidence interval (CI). Variables yielding *p *values of less than or equal to 0.20 in the bivariate analyses were entered into a multiple logistic regression model in which ICU mortality was the outcome of interest. The two episodes from the patient with recurrent PCP were treated as independent cases. A *p *value of less than 0.05 was considered statistically significant. The statistical analysis was performed using Statview5.0 (Abacus concepts, Berkeley, CA, USA) and SAS9.1 (SAS Institute Inc., Cary, NC, USA) software.

## Results

### Main characteristics of patients at admission to the intensive care unit

From January 1993 to December 2006, we identified 72 PCP patients (73 cases) admitted to our ICU (45 HIV-positive and 27 HIV-negative patients) (Table 1). HIV-positive patients were significantly younger than HIV-negative patients (37 [23 to 56] versus 64 [18 to 82] years, respectively). One HIV-positive patient suffered from recurrent PCP. The duration of symptoms before admission to the ICU was shorter in the HIV-negative than in the HIV-positive patients. Two HIV-negative patients suffered from lung fibrosis and one HIV-negative patient from sarcoidosis with pulmonary lesions. In the other patients, chronic pulmonary disease, including chronic obstructive pulmonary disease, was not reported. Chronic renal failure was reported in three HIV-negative patients and in no HIV-positive patients.

**Table 1:** Main characteristics of the patients at admission

	HIV-negative cases	HIV-positive cases
	n = 27	n = 46

Gender, male/female	11/16	34/12^a^
Age, years	64 (18–82)	37 (23–56)^a^
Duration of symptoms before ICU, days	10 (4–45)	18 (4–90)^a^
Simplified Acute Physiology Score II	38 (13–90)	27 (11–112)^a^
Respiratory rate, breaths per minute	32 (16–41)	32 (20–48)
PaO_2_/FiO_2 _ratio, mm Hg	160 (61–322)	183 (38–380)
PaCO_2_, mm Hg	34 (26–49)	35 (16–60)
Circulatory failure, number	7 (26)	7 (15)
Blood lactate, mmol/L	1.8 (0.6–16.4)	1.4 (0.2–15)

Values are expressed as median (range) or as absolute value (percentage). ^a^*P *< 0.05 versus HIV-negative cases. ICU, intensive care unit; PaCO_2_, arterial partial pressure of carbon dioxide; PaO_2_/FiO_2_, arterial partial pressure of oxygen/fraction of inspired oxygen.

### *P. jiroveci *pneumonia diagnosis

Bronchoalveolar lavage showed a higher count of neutrophils and a lower density of *P. jiroveci *in HIV-negative patients. Immunofluorescence was positive in all patients. Staining performed on bronchoalveolar lavage specimens was positive in 86% of HIV-negative cases and in 58% of HIV-positive cases (*p *= 0.46) (Table 2).

**Table 2:** Microbiological diagnosis

	HIV-negative cases	HIV-positive cases
	n = 27	n = 46

Method of diagnosis, number (percentage of cases)		
BAL	24 (89)	42 (92)
Positive at staining	14	36
Positive at immunofluorescence	24	42
Induced sputum	1 (4)	2 (4)
Positive at staining	0	2
Positive at immunofluorescence	1	2
Tracheal aspiration	2 (7)	2 (4)
Positive at staining	0	2
Positive at immunofluorescence	2	2
Density of *Pneumocystis jiroveci *on the BAL fluid^a^, percentage of all BAL		
'Many'	35%	81%
'Few'	65%	19%
Neutrophil count on the BAL, cells per microliter, median (range)	65,475 (6,000–733,500)	24,750 (320–480,000)
Other pathogens identified by BAL, number		
Cytomegalovirus	1	4
*Streptococcus pneumoniae*	2	1
*Pseudomonas aeruginosa*	0	1
*Cryptococcus neoformans*	0	1

^a^The density of *P. jiroveci *organisms was graded as 'many' when foamy alveolar casts were easily visualized on all slides and as 'few' when foamy alveolar casts were not individualized at first-glance evaluation. BAL, bronchoalveolar lavage.

### Yearly incidence of intensive care unit admissions for *P. jiroveci *pneumonia in HIV-negative and HIV-positive patients

**Figure 1:**
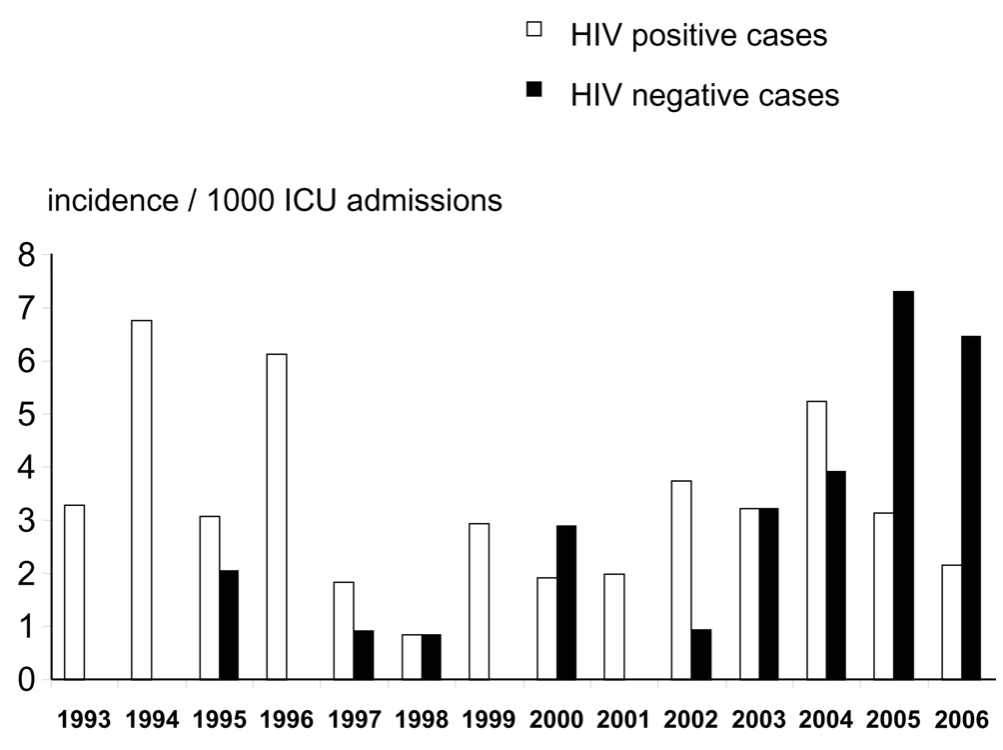
Yearly proportion among all admissions in the intensive care unit (ICU) of cases with *Pneumocystis *pneumonia in patients infected (HIV-positive) and not infected (HIV-negative) with HIV.

The yearly incidence of ICU admissions for PCP in HIV-negative and HIV-positive patients is depicted in Figure 1. The proportion of HIV-positive cases admitted for PCP among all admissions to the ICU was not correlated with time (*p *= 0.40). By contrast, the proportion of HIV-negative cases admitted for PCP among all admissions to the ICU was significantly and positively correlated with time (*r *= 0.77, *p *< 0.01). Among all admissions for PCP to the ICU, the proportion of HIV-negative cases increased from 0% in 1993 to 75% in 2006 (Figure 1).

### Immunosuppressive condition associated with *P. jiroveci *pneumonia-induced acute respiratory failure

All but one of the 27 HIV-negative patients were receiving corticosteroids at the time of admission (Table 3). The patient who was no longer undergoing steroid treatment at the time of admission had received chemotherapy following autologous bone marrow transplantation. In renal transplant recipients, the time between transplantation and PCP diagnosis was 70 (5 to 144) months. No HIV-negative patient was neutropenic, and the blood lymphocyte count was less than or equal to 1,000 cells per microliter in 17 patients. In the 10 renal transplant recipients, a chemoprophylaxis by trimethoprim-sulfamethoxazole had been administered for 1 month after transplantation but had been interrupted later. The peripheral CD4 count was available in 6 HIV-negative patients and was 244 (32 to 699) cells per microliter. It was higher than 300 cells per microliter in 3 of these 6 patients.

**Table 3:** Immunosuppressive condition associated with *Pneumocystis *pneumonia in patients who were not infected with HIV (n = 27)

Renal transplant, number	10
Chronic inflammatory disease, number	6
Rheumatoid arthritis, number	3
Dermatomyositis, number	1
Wegener disease, number	1
Temporal arteritis, number	1
Solid tumor, number	4
Hematologic malignant disorder, number	4
Chronic lymphoid leukemia, number	1
Acute lymphoid leukemia, number	1
Non-Hodgkin lymphoma, number	2
Lung fibrosis, number	2
Alcoholic hepatitis, number	1
Previous corticosteroid therapy, number	26
Duration at the time of admission, months	10 (1–144)
Daily dose, mg prednisone equivalent	13 (5–240)
Patients with a daily dose of ≤ 15 mg prednisone equivalent, number	13
Other immunosuppressive therapy, number	23
Methotrexate, number	7
Mycophenolate mofetil, number	6
Tacrolimus, number	5
Cyclosporine, number	2
Cyclophosphamide, number	2
Azathioprine, number	1
Other cancer chemotherapy, number	4
Neutrophil count, cells per microliter	670 (1,160–22,200)
Lymphocyte count^a^, cells per microliter	730 (130–3,090)

Values are expressed as median (range) or as absolute value. ^a^The lymphocyte count is reported on 24 patients after excluding 3 patients with lymphoid leukemia. The neutrophil count is reported on the whole population of HIV-negative patients (n = 27).

PCP was the first manifestation of HIV infection and revealed the HIV infection in 27 of the 46 HIV-positive cases. In 3 cases receiving pentamidine, PCP occurred during ongoing prophylaxis. No HIV-positive patient was receiving trimethoprim-sulfamethoxazole at the time of admission. The peripheral CD4 count was 21 (2 to 303) cells per microliter in this population (n = 46).

### Medications and renal replacement therapy

All patients received trimethoprim (20 mg/kg per day)-sulfamethoxazole. The time between the diagnosis of PCP and the start of appropriate treatment was not statistically different between HIV and non-HIV patients (0 [-4 to 5] days versus 0 [-2 to 3] days, respectively). In two HIV-positive patients, a skin rash was attributed to trimethoprim-sufamethoxazole and the treatment was replaced by atovaquone. Corticosteroid treatment was administered as an adjunctive therapy in all HIV-positive and HIV-negative cases (methylprednisolone 240 mg/day for 3 days, 120 mg/day for 3 days, 60 mg/day for 3 days, or until an antibacterial antibiotic was stopped [16]). Two HIV-positive patients were receiving an active antiretroviral therapy at the time of admission. It was interrupted during the ICU stay. A significantly greater proportion of HIV-negative patients required renal replacement therapy (27% versus 8%).

### Ventilatory support

A similar proportion of HIV-negative and HIV-positive cases required ventilation assistance (78% versus 61%, respectively). This proportion was not statistically correlated with time either in HIV-negative patients (*p *= 0.22) or in HIV-positive patients (*p *= 0.31). In ventilated patients, NIMV was the first-line mode of ventilation in a similar proportion of HIV-negative and HIV-positive cases (66% versus 54%, respectively; *p *= 0.79). NIMV failed in a higher proportion of HIV-negative cases compared with HIV-positive cases (71% versus 13%; *p *= 0.005).

When invasive ventilation was used, the proportion of days on ventilation during which the patient received a positive end-expiratory pressure of greater than 5 cm H_2_O was lower for HIV-positive cases (70% [3% to 100%]) compared with HIV-negative patients (90% [70% to 100%]; *p *= 0.04). In addition, the proportion of days on ventilation during which the patient received an FiO_2 _of greater than 60% was lower for HIV-positive patients (45% [6% to 100%]) compared with HIV-negative patients (100% [70% to 100%]; *p *= 0.03). In patients with acute lung injury/acute respiratory distress syndrome, tidal volume was not correlated with time either in HIV-negative patients (*p *= 0.83) or in HIV-positive patients (*p *= 0.50).

### Pneumothorax

A pneumothorax occurred in a similar proportion of HIV-positive and HIV-negative cases (Table 4). Considering the whole population, the mortality rate in patients with a pneumothorax was 58%.

**Table 4:** Outcome

	HIV-negative cases	HIV-positive cases
	n = 27	n = 46

Occurrence of ALI/ARDS, number (percentage of all cases)	16 (59)	14 (30)^a^
Occurrence of pneumothorax, number (percentage of all cases)	5 (19)	7 (15)
Occurrence of ventilator-assisted pneumonia, number (percentage of ventilated cases)	5 (26)	5 (14)
Days alive with mechanical ventilation, median (range)	9 (2–75)	9 (1–60)
ICU length of stay in days, median (range)	10 (4–131)	6 (1–93)
In-ICU mortality, number (percentage of all cases)	13 (48)	8 (17)^a^
28-day mortality, number (percentage of all cases)	14 (52)	12 (26)^a^
90-day mortality, number (percentage of all cases)	16 (59)	14 (30)^a^

^a^*P *< 0.05 versus HIV-negative cases. ALI/ARDS, acute lung injury/acute respiratory distress syndrome; ICU, intensive care unit.

### Ventilator-associated pneumonia

Ventilator-associated pneumonia occurred in a similar proportion of HIV-negative and HIV-positive cases (Table 4). It was related to *Pseudomonas aeruginosa *in 7 patients, *Klebsiella pneumoniae *in 2 patients, and *Enterobacter cloacae *in 1 patient. It occurred after NIMV failed in 4 HIV-negative cases and in 1 HIV-positive case and after first-line endotracheal intubation in 1 HIV-negative case and in 3 HIV-positive cases.

### Mortality

Mortality was higher in HIV-negative than in HIV-positive cases (Table 4). Mortality was not correlated with time for HIV-negative patients (*p *= 0.17) or for HIV-positive patients (*p *= 0.95). When ventilation was needed, the ICU mortality rates were 62% in HIV-negative and 29% in HIV-positive cases (*p *= 0.002). Mortality in patients requiring mechanical ventilation was not correlated with time either in HIV-negative patients (*p *= 0.10) or in HIV-positive patients (*p *= 0.52). When NIMV failed, mortality rates were 80% in HIV-negative and 0% in HIV-positive cases. Predictors of ICU mortality at bivariate analysis are presented in Table 2. Multivariate analysis revealed that the negative HIV status (OR 3.73, 95% CI 1.10 to 12.60) and SAPS II (OR 1.07, 95% CI 1.02 to 1.12) were independently associated with increased ICU mortality (Table 5).

**Table 5:** Bivariate analysis: predictors of intensive care unit mortality

	Survivors	Non-survivors	*P *value
	n = 52	n = 21	

HIV-positive status, number (percentage)	38 (73)	8 (38)	0.01
Age in years, median (range)	39 (18 to 80)	52 (25 to 82)	0.01
Simplified Acute Physiology Score II, median (range)	28 (6 to 56)	56 (22 to 112)	<0.01
Occurrence of pneumothorax, number (percentage)	5 (10)	7 (33)	<0.01
Occurrence of renal failure, number (percentage)	5 (10)	4 (19)	0.27
Hemodynamic failure at admission, number (percentage)	17 (33)	11 (52)	0.12
Non-invasive mechanical ventilation failure, number (percentage)	4 (8)	8 (38)	<0.01
Time between diagnosis of PCP and appropriate therapy in days, median (range)	0 (-2 to 3)	0 (-1 to 4)	0.50
PaO_2_/FiO_2 _ratio, mm Hg, median (range)	179 (38 to 322)	154 (59 to 377)	0.20

PaO_2_/FiO_2_, arterial partial pressure of oxygen/fraction of inspired oxygen; PCP, *Pneumocystis jiroveci *pneumonia.

## Discussion

This retrospective study demonstrates that the incidence of PCP requiring ICU admission has increased in HIV-negative patients at our institution during the period of 1993 to 2006. As compared with HIV-positive cases, non-HIV patients had a worse course of the disease in the ICU. ICU mortality was higher in HIV-negative than in HIV-positive patients. Importantly, first-line NIMV failed in a very large proportion of HIV-negative patients.

HIV-negative status, which is known to be associated with an increased mortality during PCP compared with HIV-positive status [3,5,6,17-19], maintained this grim prognostic value for the critical forms of the disease. This difference in mortality might be related to the underlying condition rather than to the HIV-negative status *per se*. Not only the mortality but also the proportion of ventilated days spent with high levels of positive end-expiratory pressure and FiO_2 _were higher in HIV-negative compared with HIV-positive patients. The higher neutrophil count observed in the bronchoalveolar lavage of HIV-negative patients suggests that the PCP-related lung injury was more severe in HIV-negative subjects. Even though we could not assess whether baseline differences in age and chronic lung condition influenced this finding, it suggested that the lung injury was different between HIV-positive and HIV-negative patients. This had important implications in terms of ventilatory support modalities.

Indeed, one of the most striking results concerned the description of the ventilatory support depending on the HIV status, a comparison that has not been performed to date. NIMV was chosen as first-line therapy in a similar proportion of HIV-negative and HIV-positive patients. However, in HIV-negative patients, NIMV failed in 71% of cases compared with failure in 13% of HIV-positive patients, suggesting that the severity of PCP-related lung injury was tremendously higher in HIV-negative patients. By contrast, in the 29% of HIV-negative patients in whom NIMV succeeded, NIMV avoided tracheal intubation and its associated poor prognosis. In this regard, our results are in full accordance with the well-known benefit of NIMV in different populations of immunosuppressed patients with other causes of respiratory failure [20,21]. The retrospective nature of our study, with no standardized modality for ventilatory support, does not allow for conclusions concerning the respective indication of both techniques in this particular population. Rather, the clinical implication of our study is that when NIMV is attempted in a patient with PCP-related acute respiratory failure, the clinician should consider an HIV-positive and an HIV-negative patient with PCP-induced respiratory failure very differently, with a more vigilant watch on HIV-negative cases with NIMV support. This is emphasized by the fact that 80% of HIV-negative patients with NIMV failure died, confirming that in this setting as in others [22,23], NIMV failure is associated with a poor prognosis. Importantly, the fact that SAPS II was independently associated with mortality suggests that the worse prognosis of mechanical ventilation was related to a poorer condition of HIV-negative patients at the time of admission. In line with this, a limitation of the present study is that the need for mechanical ventilation was not adjusted for the baseline differences in age and chronic lung condition in HIV-positive and HIV-negative patients.

By contrast, in HIV-positive patients, NIMV succeeded in a large majority of cases, according to the less severe lung alteration by PCP in these patients. Furthermore, when NIMV did fail in HIV-positive patients, the patient survived, reinforcing the evidence of the benefit that could arise from NIMV in severe AIDS-related PCP with acute respiratory failure [9,24,25]. The increased incidence of HIV-negative patients with PCP-induced acute respiratory failure observed in our series confirms previous reports [3-5]. This increase possibly was related to a higher prevalence of immunosuppressed patients in our institution, as suggested by the increase in the cohort of transplant recipients. Other factors like heightened awareness for pursuing the diagnosis of PCP, increased familiarity with diagnostic staining methods and detection, and so on also could have accounted for that increase. Importantly, a high proportion of HIV-negative patients had received corticosteroids at a daily dose of less than 15 mg prednisone prior to admission but the majority of these patients were concomitantly exposed to another immunosuppressive therapy. These results raise serious concerns about the appropriateness of guidelines for PCP prophylaxis in HIV-negative immunosuppressed patients, and studies focusing on this question should be recommended. It is noteworthy that in half of the HIV-negative patients in whom it was performed, the CD4 count was higher than 300 cells per microliter, the cutoff value that has been proposed to detect HIV-negative patients at risk for PCP [26]. In line with this, a recent meta-analysis of studies conducted in bone marrow transplant recipients suggested that a clinical PCP risk threshold rather than a CD count threshold should be used for deciding to administer prophylaxis against PCP in that population [27].

As reported before [4,6], symptoms were more acute in HIV-negative than HIV-positive patients. The density of *P. jiroveci *in the bronchoalveolar lavage specimens was lower in non-AIDS patients, which is well known [18,28,29]. Interestingly, the standard staining methods failed to detect *P. jiroveci *in a large proportion of HIV-negative patients. As an important clinical implication, immunofluorescence should be systematically performed in non-AIDS patients with suspected PCP. Twenty-six percent of HIV-negative patients presented with acute circulatory failure at ICU admission, confirming that the PCP-related systemic inflammatory response syndrome could impair hemodynamics similar to viral or bacterial infections [30].

We acknowledge several limitations to our study. First, it reflects the experience of a single center. Second, the study is retrospective and neither the choice of the ventilation support nor the modalities of this support were chosen according to predetermined guidelines. Third, the study period was long and the modalities of critical care may have changed over the years, especially for the use of NIMV or HIV management. It is difficult to say what influence this had on the prognosis of PCP, but most likely it did not alter the relevance of the comparison between HIV-negative and HIV-positive cases.

## Conclusion

The incidence of PCP in HIV-negative patients in our unit increased from 1993 to 2006. The course of the disease and the outcome were worse in HIV-negative patients than in HIV-positive patients. Importantly, despite its benefit, NIMV often failed in HIV-negative patients and should be cautiously monitored in this population.

## Key messages

• The incidence of *Pneumocystis *pneumonia requiring critical care increased in patients with no HIV infection in our unit from 1993 to 2006.

• Although non-invasive ventilation was required in a similar proportion of patients with and without HIV infection, non-invasive ventilation failed in a very high proportion of HIV-negative patients but succeeded in the vast majority of HIV-positive patients.

• The HIV-negative status was an independent predictor of mortality of patients with critical *Pneumocystis *pneumonia.

## Abbreviations

CI = confidence interval; FiO_2 _= fraction of inspired oxygen; ICU = intensive care unit; NIMV = non-invasive mechanical ventilation; OR = odds ratio; PCP = *Pneumocystis jiroveci *pneumonia; SAPS = Simplified Acute Physiology Score.

## Competing interests

The authors declare that they have no competing interests.

## Authors' contributions

XM conceived the study, contributed to the collection of data, performed analysis and interpretation of data, and drafted the manuscript. EV-P conceived the study, performed the collection of data, and contributed to the analysis and interpretation of data and to the drafting of the manuscript. XM and EV-P contributed equally to this study. DO contributed to the collection, analysis, and interpretation of data and to the drafting of the manuscript. OH contributed to the collection of data. AD, CG, CM, and PB were involved in drafting the manuscript or revising it for intellectual content. CR conceived the study, participated in its design and coordination, and helped to draft the manuscript. All authors read and approved the final manuscript.
